# ‘Cinemadness’: In search of sanity in films

**DOI:** 10.4103/0019-5545.58287

**Published:** 2009

**Authors:** G. Swaminath, Ajit Bhide

**Affiliations:** Dr. B. R. Ambedkar Medical College, Kadugondanahalli, Bangalore - 560 045, Karnataka, India; 1 Department of Psychiatry, St. Martha's Hospital, Bangalore - 560 001, India

## THE CAMERA NEVER LIES, BUT WHAT ABOUT THE FILM MAKER?

Dr. Ashok Pai, a veteran psychiatrist and a film script writer and producer, from the once sleepy town of Shimoga in Karnataka, made some astute observations on the strange story-telling in some highly successful ‘action films’.

The secret agent enters the villain's den and finds himself in a dingy warehouse stacked with gunnysacks full of a whitish powder. Dipping his finger into one of them, he licks it and exclaims, “Uranium!” This fills the minds of audience with horror about the audacity of the unseen villain and his capacity to amass ingredients of mass destruction. Very few among the audience reflect on the important scientific issues related to this scene. For example, can uranium, a dangerous radioactive chemical, be stored in gunnysacks? Will not such storage create havoc across miles, most importantly for the nearby goons on whose strength the villain rules? Will any intelligent person, especially a secret agent, ever put his finger into an unknown substance and lick it as a means of identification? And finally, how come a radioactive element when licked does not produce devastating effects on the hero?

Cinema in general and Indian cinema in particular has a fantastic disconnectedness from reality.

Are there really beautiful 20-year-old blonde nuclear scientists wandering in bikinis in their labs?[1] Is there a 20-year-old petite neurosurgeon as portrayed in the movie *Kambakt Ishq?* Do terrorists really build bombs with convenient displays that inform us how much time we have to deactivate the contrivance?[1] Do men and women in Indian villages really run around trees, singing songs of undying love with a full orchestra playing in the background? Are the police always corrupt and incompetent?[1]

These questions never arise in the audience's mind as the sole purpose of the audience in viewing the film is to drown in fantasy while being entertained. Unfortunately, such crass fare is served to pass for ‘wholesome entertainment.’ Many, who understand the irrationalities of a scene, are willing to suspend logical judgment and even accept it as artistic license. The rest, who fail to understand this, take back the message that uranium can be stored in gunnysacks and such associated irrationalities. This shapes their knowledge, their attitude and perception of issues related to uranium. The power of the medium can never be overestimated.

The passive acceptance of irrational ideas, as in the scene described above, does not necessarily affect society at large in any measurable way, given the remoteness of the chances of the average moviegoer coming into contact with anything related to nuclear physics. However, the same stance cannot be taken with regard to sensitive issues, such as mental illness. Owing to their sheer novelty and the mystic element, psychological issues, mental illness and the mentally ill occupy an important place in the minds of film makers. Films on issues related to mental illness have been box office hits, and we would reckon that they feature at least among the top 50 films of all time in any language. This repeated portrayal of the mentally ill and mental illness with the usual distortions has contributed a lot to the stigma and burden which the mentally ill and their caretakers have to bear.

## TIME TO CHANGE

It is now time to change. And heralding this is a remarkable effort by a leading psychiatrist, Dr. Peter Byrne, a researcher in the field of stigma of mental illness; and a film expert, Sue Baker, who have started *Time to Change,* an advocacy group. Their slogan is, “Let's end mental health discrimination.”[2] Dr. Byrne has brought out a report, *Screening Madness,* which analyzes the contributions of cinema in influencing public perception and attitudes positively or otherwise.[2] The report states that characters with mental health problems are being depicted as “more demonic and crueler than at any time in movie history.”[3] There are of course notable exceptions that many of us will recall: *Girl Interrupted* (borderline personality disorder), *Devarai* (Marathi), *A Beautiful Mind,* (schizophrenia) and *Khamoshi* (Hindi, transference-related problems).

Few mentally ill individuals will openly disclose their mental health problems, as stigma drives any chance of healthy discussion underground. There is prejudice against the mentally ill, with them being stereotyped as ‘mad’ and expected to be violent.[2]

Time to change undertook a YouGov survey with more than 2000 members of the public to assess their attitudes to film and mental health.[2] Half of the respondents indicated that they had seen violent ‘mentally ill’ characters in TV documentaries or films. A further 29% said they read in the newspapers about violence by people with mental health problems. When asked what characteristics define film characters with mental illnesses, the top three answers were violent (39%), weird (35%) and likely to kill violently (30%).

Films need drama and conflict to engage the audience, and mental illnesses and the mentally ill easily serve this purpose; the more extreme the behavior, the better. The story (if the central character is mentally ill) usually highlights dramatic breakdowns, relapses,[2] absence of adequate and good treatment, the inevitability of chronicity and finally poor outcome. The situations reach the pinnacles of comedy, violence, indulgence or pathos.[2] If the drama is resolved, this happens quickly and in an idealized way: Illness being cured by the tumbling out of dark secrets (Equus) or by falling in love.

The report *Screening Madness* identifies popular films as a reservoir of prejudice, ignorance and fear that perpetuates damaging stereotypes of people with mental health problems. It reveals the evidence that links powerful negative images with public prejudice.[2]

The report[2] identifies four stereotypes of people with mental health problems in mainstream cinema: i) The ‘comedy’ stereotype, notable examples being *High Anxiety* (1977), *Analyse This* (1999), *Analyse That* (2002), and *Me, Myself and Irene* (2000); ii) ‘faking and indulgent’ stereotype, examples being *King Lear* (1971/1999), *One Flew Over the Cuckoo's Nest* (1975) and *Primal Fear* (1996); iii) ‘pity’ stereotype, depicted in *One Flew Over the Cuckoo's Nest* (1975), *What Dreams May Come* (1998); and iv) ‘violence’ stereotype, which is seen in two ways: psychosis causing violence as seen in *Taxi Driver* (1975), *Asylum* (2005); and the other, ‘psychokiller’ films, the examples being *Psycho* (1960), *Friday the Thirteenth* (1980) and *Silence of the Lambs* (1991), many of which have had multiple sequels and remakes, indicating their money-spinning abilities.

### Sense, nonsense and non-science

Film historians have documented racist and homophobic stereotypes in films right up to the 1970s. On account of changes in society's sensibilities, today's releases contain watered-down versions of these. The same is true for homosexual stereotypes; the gay man no longer has to be the effete limp-wristed pansy. However, mental health stereotypes have not changed over a century of cinema.[2] If anything, the comedy is crueler; and the deranged psychokiller, even more demonic than earlier prototypes.[3]

Comedy and satire have been used to great cinematic effect to challenge public prejudice, both against racism and homophobia.[2] This is in keeping with the society's growing awareness about, and improved attitudes towards, these issues. It isn't just that film makers seem unable to understand characters with mental health problems; often the comedy is based on that lack of understanding.[2]

To cinema with powerful psychiatric melodramas, we respond with neither laughter nor fear, but pity for the bewildered mental patient as victim.[2] The supporting players of *One Flew Over the Cuckoo's Nest* (or of many of the Hollywood and Bollywood movies) were weak, stammering, easily controlled and of bizarre appearance.[2] There seems to be no distinction between intellectual disability and psychotic disorder, and the character ends up with a bizarre depiction, like the patient making strange body movements.[4] Sometimes, the psychiatrists are buffoonish and end up falling in love with patients,[4] breaching confidentiality, prescribing Electro convulsive therapy (ECT)[4,5] in a sinister manner or offering weird advice.[4]

Most of the film makers have commercial interest and do not worry about clinical accuracy and contemporary treatment principles. While many modern films (*Twelve Monkeys*, 1995) accurately depict the under-funding of psychiatric units and treatments (the Chief Resident tells her doctors to “evaluate, medicate and vacate” all new admissions), images from these melodramas show asylums as human zoos.[2] The important point here is that no disadvantaged group ever achieved equality by getting the public to feel sorry for its members. To beat stigma against people with mental health problems, we need parity, not pity.[2]

### Onus on us

Members of our profession need to be vigilant in guiding the gullible public when they are forced to view execrable matter — all in the name of entertainment. Dr. Harish Shetty's exemplary initiative with an NGO, Maitri, is worth emulation — the initiative in exposing through the Human Rights Commission (application no. 964/13, 2005-2006) the fraudulent depiction of both illness and treatment in cinema. The media cells of professional bodies like the IPS should lead the way in this endeavor.

There have been some eye-opening works on films and psychiatry. Most recently, Prof. Dinesh Bhugra's *Mad Tales of Bollywood*[6] has exhaustively studied mental disorder in Hindi cinema. Among other seminal tomes are the scholarly *Psychoanalysis and Film*[7] and *Psychiatry and the Cinema*[8] by Prof. Glen Gabbard.

Film as a medium should be used to advantage to dispel the stigma associated with psychiatric disorders. Care should be taken to ensure that only scientifically sound messages are conveyed to the audience[9] and for this, it is essential to have a censor board that is sensitive and well informed. The dissemination of erroneous messages relating to mental disorders through cinema only aggravates the longstanding problem of stigma associated with such disorders.

We live in an age where we are seeing more psychiatrists in cinema than ever before. The complexities of modern life have made at least one visit to the psychiatrist essential in at least every third film! In fact, the number of feature films without a psychiatrist or a huge element of fantasy is practically negligible, but that is another story.
